# The UK kidney donor risk index poorly predicts long-term transplant survival in paediatric kidney transplant recipients

**DOI:** 10.3389/fimmu.2023.1207145

**Published:** 2023-06-02

**Authors:** Jon Jin Kim, Rebecca M. K. Curtis, Ben Reynolds, Stephen D. Marks, Martin Drage, Vasilis Kosmoliaptsis, Jan Dudley, Alun Williams

**Affiliations:** ^1^ Department of Surgery, University of Cambridge, Cambridge, United Kingdom; ^2^ Department of Paediatric Nephrology, Nottingham University Hospitals, Nottingham, United Kingdom; ^3^ Statistics and Clinical Research, NHS Blood and Transplant, Bristol, United Kingdom; ^4^ Department of Paediatric Nephrology, Royal Hospital for Children, Glasgow, United Kingdom; ^5^ Department of Paediatric Nephrology, Great Ormond Street Hospital for Children NHS Foundation Trust, London, United Kingdom; ^6^ NIHR Great Ormond Street Hospital Biomedical Research Centre, University College London Great Ormond Street Institute of Child Health, London, United Kingdom; ^7^ NIHR Blood and Transplant Research Unit in Organ Donation and Transplantation, University of Cambridge, Cambridge, United Kingdom; ^8^ Department of Paediatric Nephrology, Bristol Children’s Hospital, Bristol, United Kingdom

**Keywords:** paediatric kidney transplantation, kidney allocation, donor quality, donor age, donor risk index, HLA mismatching, prediction model

## Abstract

**Background:**

The UK kidney offering scheme introduced a kidney donor risk index (UK-KDRI) to improve the utility of deceased-donor kidney allocations. The UK-KDRI was derived using adult donor and recipient data. We assessed this in a paediatric cohort from the UK transplant registry.

**Methods:**

We performed Cox survival analysis on first kidney-only deceased brain-dead transplants in paediatric (<18 years) recipients from 2000-2014. The primary outcome was death-censored allograft survival >30 days post-transplant. The main study variable was UK-KDRI derived from seven donor risk-factors, categorised into four groups (D1-low risk, D2, D3 and D4-highest risk). Follow-up ended on 31-December-2021.

**Results:**

319/908 patients experienced transplant loss with rejection as the main cause (55%). The majority of paediatric patients received donors from D1 donors (64%). There was an increase in D2-4 donors during the study period, whilst the level of HLA mismatching improved. The KDRI was not associated with allograft failure. In multi-variate analysis, increasing recipient age [adjusted HR and 95%CI: 1.05(1.03-1.08) per-year, p<0.001], recipient minority ethnic group [1.28(1.01-1.63), p<0.05), dialysis before transplant [1.38(1.04-1.81), p<0.005], donor height [0.99 (0.98-1.00) per centimetre, p<0.05] and level of HLA mismatch [Level 3: 1.92(1.19-3.11); Level 4: 2.40(1.26-4.58) versus Level 1, p<0.01] were associated with worse outcomes. Patients with Level 1 and 2 HLA mismatches (0 DR +0/1 B mismatch) had median graft survival >17 years regardless of UK-KDRI groups. Increasing donor age was marginally associated with worse allograft survival [1.01 (1.00-1.01) per year, p=0.05].

**Summary:**

Adult donor risk scores were not associated with long-term allograft survival in paediatric patients. The level of HLA mismatch had the most profound effect on survival. Risk models based on adult data alone may not have the same validity for paediatric patients and therefore all age-groups should be included in future risk prediction models.

## Introduction

Children and young adults with end-stage kidney disease (ESKD) require transplants which last their lifetime (1, 2). Young adults with childhood onset ESKD are reported to have a life expectancy of 38 years if treated with dialysis and 63 years if they receive a kidney transplant that functions well (3). We recently reported that 42% of first paediatric kidney transplants are still functioning at 20 years - 40% following donation after brain death (DBD) and 49% for living donor kidney transplants (4). Survival of DBD transplants has significantly improved over time, mainly due to improvements in 12-month survival rates, rising from 72% (1987-1991) to 98% (2012-2016). Beyond the first year, the rate of graft attrition has remained constant.

In order to improve deceased donor transplant survival, national kidney allocation policies have developed strategies for matching donors to recipients, broadly based on kidney donor ‘quality’ factors and donor-recipient human leucocyte antigen (HLA) mismatching (5–8). In the United States (USA), the Kidney Donor Risk Index (KDRI) was developed based on ten donor variables and implemented in 2012 (9). The donors with the lowest (best) 35% KDRI were prioritised to paediatric patients (10). The USA KDRI model performs less well for paediatric donors as it over-estimates the risk from smaller sized donors with otherwise well-functioning kidneys (10, 11). In the United Kingdom (UK), HLA mismatching was introduced in 2006 through categorising HLA mismatches into 4 levels (4). This was followed in 2019 with a UK-population based KDRI of seven donor variables (12). Both indices were trained and tested on adult donor and recipient populations.

The UK-KDRI is currently used to allocate a subset of points in the kidney offering scheme (13). The UK-KDRI is grouped into four quartiles - D1 (best), D2, D3 and D4 (worst) and a separate recipient risk index (RRI) is calculated and grouped into four quartiles - R1 (best), R2, R3 and R4 (worst). Of note, KDRI is modelled on (death censored) allograft survival and RRI is modelled on all-cause transplant survival which includes patient death. Paediatric recipients score in the R1 group and receive more allocation points for D1 donors, with decreasing points from D1 to D4. In this study, we aimed to apply and validate the UK-KDRI as a risk factor for death-censored allograft failure in UK paediatric kidney transplant recipients following donation after brain death (DBD). As a secondary objective, we re-analysed individual donor and recipient risk factors in our cohort with long term follow-up of up to 21 years.

## Methods

### Patient population

Data were obtained from the UK Transplant Registry, held by NHS Blood and Transplant (NHSBT). NHSBT mandates collection of transplant activity and outcomes. The dataset was de-identified by NHSBT prior to use for research. All first DBD kidney only transplants in paediatric recipients (<18 years of age) performed between 2000-2014 (inclusive) were included. This study period was chosen to represent contemporaneous management and enable long-term follow-up. Some donor variables were also less complete pre-2000. Last follow-up was taken on 31 December 2021. Transplants from donors following circulatory death (DCD, n=22) were excluded as their usage was not uniform during the earlier years (4). We also excluded multi-organ transplants, transplants from dual *en-bloc* kidneys and re-transplants. Cause of allograft failure was categorised using the primary cause reported to NHSBT, which of note, does not differentiate between rejection subtypes.

During the study period, the UK kidney allocation scheme was updated in 2006. HLA matching was prioritised for paediatric recipients and four levels of HLA mismatching were introduced: Level 1 - 000 HLA-A,B, DR mismatch, Level 2 - 0 DR +0/1 B mismatch (Level 1 and 2 were classed as favourable), Level 3 - 0 DR + 2B mismatch OR 1 DR + 0/1 B mismatch, Level 4 - 1 DR + 2 B mismatch OR 2 DR mismatch (least favourable) (4). Also, paediatric recipients loss priority for paediatric donor kidneys but had increased access to adult donor kidneys up to the age of 50 years (4).

### UK KDRI

The KDRI score is calculated using the following formula (14):


$$\begin{array}{l} \text{UK KDRI}=\exp\;\{\left(0.023*\left(\text{donor age},\text{yrs}-50\right)\right)+\\ \;\left(-0.152*\left(\left(\text{donor height},\text{cm}-170\right)/10\right)\right)+\\ \;0.149*\text{donor hypertension},\text{yes}/\text{no})+\\ \;\left(-0.184*\text{female donor},\text{yes}/\text{no}\right)+\\ \;\left(0.190*\text{donor CMV status},\text{yes}/\text{no}\right)+\\ \;\left(-0.023*\left(\left(\text{offer eGFR}-90\right)/10\right)\right)+\\ \;\left(0.015*\text{days in hospital}\right)\} \end{array}$$

Donor estimated glomerular filtration rate (eGFR) is calculated using the Modification of Diet in Renal Disease (MDRD) Study equation:


$$\text{offer eGFR}=186*\left({\left(\text{creatinine}/88.4\right)}^{-1.154}\right)*\left(\text{donor age},{\text{years}}^{-0.203}\right)*\left({\text{female donor}}^{0.742}\right)*\left({\text{black donor}}^{1.210}\right)$$

KDRI scores are split according to the quartiles defined in the kidney allocation scheme - D1 ≤ 0.79, D2 0.79 – 1.12, D3 1.12 – 1.50, D4 ≥1.50 (14).

In addition, we performed analysis based on the simplified, five-variable, UK Watson KDRI formula (15):


$$\begin{array}{l} \text{Watson KDRI}=\exp\{\left(-0.245*\left(\text{donor age}<40\text{years},\text{yes}/\text{no}\right)\right)+\\ \;\left(0.396*\left(\text{donor age},>60\text{years},\text{ye}s/\text{no}\right)\right)+\\ \;\left(0.265*\text{donor hypertension},\text{yes}/\text{no}\right)+\\ \;\left(0.0253*\left(\left(\text{donor weight},\text{kg}-75\right)/10\right)\right)+\\ \;\left(0.00461*\text{days in hospital}\right)+\\ \;\left(0.0465*\text{adrenaline},\text{yes}/\text{no}\right)\} \end{array}$$

### Statistical analysis

Acute kidney injury (AKI) was classified using Kidney Disease Improving Global Outcomes (KDIGO) criteria using the donor terminal creatinine and urine output in the last hour and average urine output per hour in the last 24 hours.

Data missingness: Data was complete across all variables in 60% of cases. Data for delayed graft function was unknown for 25% of cases and not analysed. Data missingness for donor factors were low (1-6.5%) [**Figures S1A, B**]. The pattern of missingness was random [**Figure S1C**]. Data on primary outcome was complete. Missing values were imputed to the median.

Survival analysis: The primary outcome was death censored graft survival. The primary exposure variable was the UK-KDRI risk group. UK-KDRI and Watson formula were also analysed as continuous scores. Of note, there were only eight patients using the Watson high risk threshold ≥1.35 (14). We further performed univariate analysis of donor, recipient and transplant factors including the year of transplant. In the final multi-variable analysis, we tested all univariate variables (without UK-KDRI) and kept variables based on forwards and backwards elimination which maximised the Akaike Information Criterion (AIC). For donor and recipient age, variables were fitted both as a linear variable and a polynomial spline model with three degrees of freedom. Proportional hazards assumption was checked using Schoenfeld residuals and was significant for recipient age - the hazard ratio declined over time. We therefore fitted a time-dependent model with recipient age divided into two time periods, 0-10 years and >10 years post-transplant. For further sensitivity analysis, we analysed graft failure with death as a competing risk using the Fine-Grey method. P-values<0.05 were deemed to be statistically significant. Analysis was performed using the survival package in R [version 3.4-0].

## Results

Between 2000-2014, 1728 primary kidney only transplants were performed in paediatric recipients (<18 years of age), of which 950 were from DBD transplants. Patient survival at 5, 10 and 15 years post-transplant was 98.3%, 96.0% and 93.7% respectively. Graft survival at 5, 10 and 15 years was 82.3%, 67.5% and 58.6% respectively. Forty-two patients had graft failure in the first 30 days post-transplant, mainly due to graft thrombosis, and were excluded from further analysis, leaving 908 patients. Characteristics of included patients are presented in **Table S1**. Patients were censored at last follow-up on 31 December 2021 and therefore, longest follow-up was 21 years. The primary outcome of death-censored allograft failure, occurred in 319/908 (35%) patients. The main cause of allograft failure was ‘rejection’ (55%) though a large percentage (37%) were classified as ‘unknown’ [**Figure S2**].

### Donor characteristics

The characteristics of deceased donors are presented in **Table 1** grouped according to the UK-KDRI risk groups. As expected, donor age increased with each group, with donors in D1 being young: inter-quartile range of 15-30 years versus D2-4: 37-49 years (p<0.001). D1 donors had higher proportions of hypertension, history of smoking and CMV positivity. Estimated GFR at time of offer was significantly higher in D1 (median 111 ml/min/1.73m^2^) versus D2-D4 (82-93 ml/min/1.73m^2^, p<0.001). As the study focused on donation following brain death, the main cause of death was intracranial haemorrhage and cardiovascular disease. In the D1 group, there was a higher proportion of donor death due to trauma. The level of HLA mismatching was better in transplants from D2-D4 donors with 10-12% transplants being Level 3-4 mismatches versus 31% of transplants from D1 donors. Though most patients received transplants from donors in the D1 group (577, 64%), a significant proportion of donors were transplanted from higher donor risk groups (D2: 230, 25%; D3: 87, 9%; D4: 14, 2%).

**Table 1:** Donor characteristics grouped by KDRI for the entire study period.

Donor characteristic	D1 (n=577)	D2 (n=230)	D3 (n=87)	D4 (n=14)	p-value
**Age (years)** **Median (Q1, Q3)**	18 (15, 30)	41 (37, 46)	45 (41, 48)	47 (47, 49)	<0.001
**Sex** **Female** **Male**	183 (32%) 394 (68%)	134 (58%) 96 (42%)	71 (82%) 16 (18%)	13 (93%) 1 (7%)	<0.001
**Blood group** **A** **B** **O** **AB**	210 (36%) 32 (5.5%) 327 (57%) 8 (1%)	81 (35%) 15 (6.6%) 132 (57%) 2 (1%)	29 (33%) 8 (9%) 50 (58%) 0 (0%)	4 (29%) 0 (0%) 10 (71%) 0 (0%)	
**Ethnicity** **White ethnic group** **Minority ethnic groups**	558 (97%) 19 (3%)	225 (98%) 5 (2%)	78 (90%) 9 (10%)	14 (100%) 0 (0%)	0.015
**Height (cm) Median (Q1, Q3)**	172 (162, 180)	169 (164, 173)	162 (157, 165)	152 (150, 158)	<0.001
**Weight (kg) Median (Q1, Q3)**	65 (55, 75)	72 (65, 80)	70 (62, 79)	60 (56, 73)	<0.001
**eGFR at offer (ml/min/1.73m^2^) Median (Q1, Q3)**	111 (90, 144)	93 (73, 111)	83 (64, 103)	82 (75, 98)	<0.001
**History of hypertension (yes)**	7 (1.2%)	31 (13%)	22 (25%)	4 (29%)	<0.001
**CMV status (positive)**	136 (24%)	97 (42%)	65 (75%)	13 (93%)	<0.001
**Hospital stay (days) Median (Q1, Q3)**	1 (1, 3)	1 (1, 3)	1 (1, 3)	1.5 (1.0, 8.7)	0.2
**Adrenaline use (yes)**	0 (0%)	0 (0%)	0 (0%)	0 (0%)	<0.001
**History of diabetes (yes)**	11 (2%)	0	0	0	0.085
**History of smoking (yes)**	185 (32%)	118 (51%)	42 (48%)	9 (64%)	<0.001
**Urine output in last hour (ml/kg/hr)**	1.7 (0.9, 3.1)	1.5 (0.8, 2.9)	1.3 (0.9, 2.6)	1.8 (1.1, 3.3)	0.2
**AKI Stage** **0** **1** **2** **3**	545 (94%) 16 (3%) 12 (2%) 4 (1%)	209 (91%) 14 (6%) 3 (1%) 4 (2%)	81 (93%) 5 (6%) 1 (1%) 0	13 (93%) 0 1 (7%) 0	0.2*
**Cause of death** **Cardiovascular or stroke** **Trauma** **Other**	272 (47%) 207 (36%) 98 (17%)	193 (84%) 22 (10%) 15 (6%)	79 (91%) 2 (2%) 6 (7%)	13 (93%) 1 (7%) 0	<0.001*
**HLA Level** **1** **2** **3** **4**	49 (8.5%) 349 (60%) 148 (26%) 31 (5.4%)	40 (17%) 164 (71%) 24 (10%) 2 (1%)	11 (13%) 66 (76%) 10 (11%) 0 (0%)	0 (0%) 14 (100%) 0 (0%) 0 (0%)	<0.001*

*comparisons between groups performed with D3 and D4 combined due to zero patients in the variable subgroups.

### Trends in donor characteristics over time

**Figure 1:**
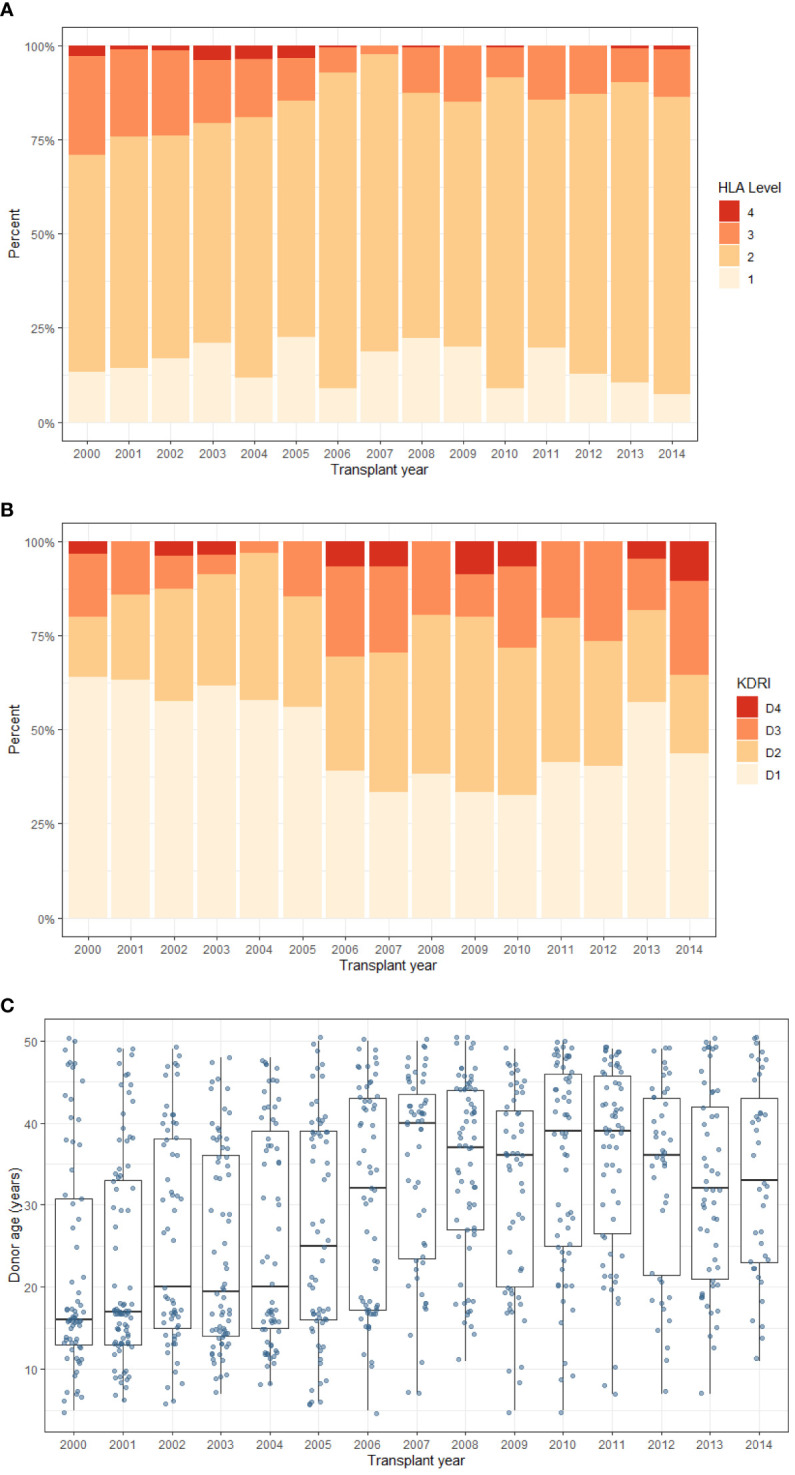
Trends in donor characteristics during the study period. Of note, the kidney offering scheme was updated in 2006 to add HLA mismatching levels (1–4) with priority to paediatric recipients. **(A)** HLA mismatch level **(B)** UK-KDRI group **(C)** Donor age (box plot represents median and inter-quartile range, and ‘whiskers’ representing range). Level 1 - 000 HLA-A,B, DR mismatch, Level 2 - 0 DR +0/1 B mismatch (Level 1 and 2 were classed as favourable), Level 3 - 0 DR + 2B mismatch OR 1 DR + 0/1 B mismatch, Level 4 - 1 DR + 2 B mismatch OR 2 DR mismatch (least favourable).

The 2006 change in the UK Kidney Offering Scheme introduced prioritisation of paediatric recipients for favourable HLA matched donors and resulted in an increase of ‘well-matched’, Level 1 and 2 mismatched kidneys [**Figure 1A**]. In addition, the proportion of donors in the D1 risk group fell from 60% pre-2006 to 40% post-2006 [**Figure 1B**]. Donor age is the main factor representing donor transplant quality and is presented in **Figure 1C**. Though there is annual variability, the median donor age increased from 18-20 years pre-2006 to 30-40 years post-2006. Nonetheless, there was a wide range of donor ages throughout the study period.

### Risk factors for death-censored allograft loss

**Figure 2:**
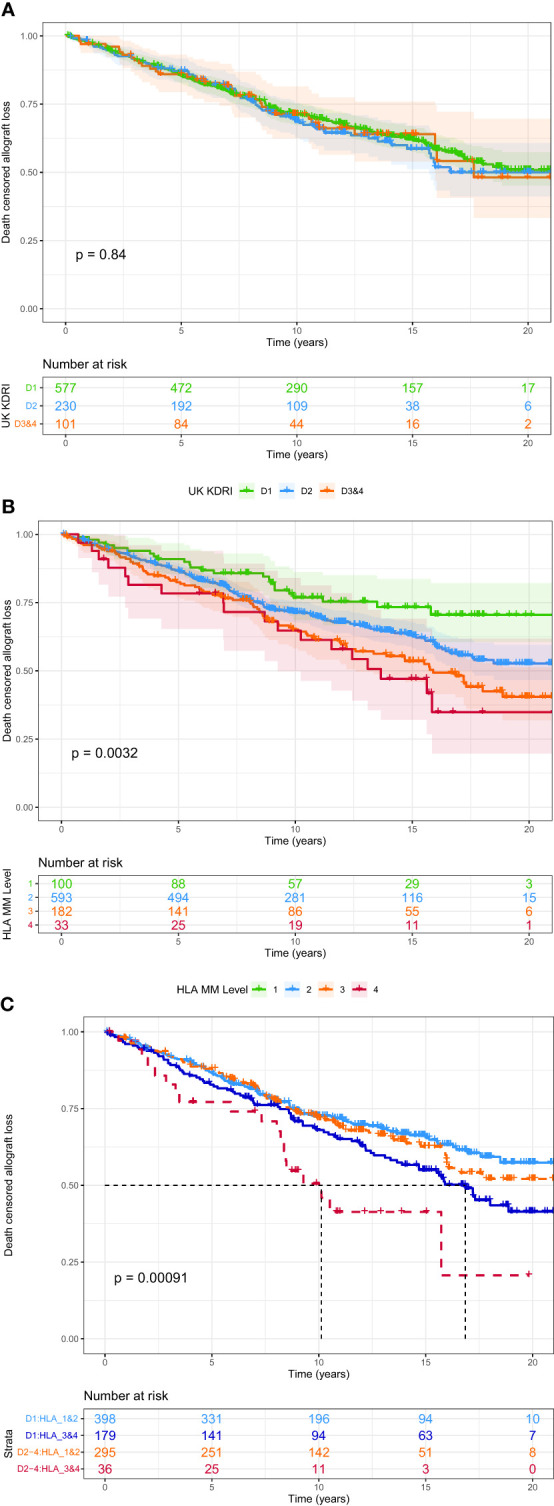
Survival analysis for all-cause allograft failure. **(A)** Kaplan Meier curve for UK KDRI groups, **(B)** Kaplan Meier curve for HLA mismatch levels, **(C)** UK KDRI D1 v D2-4 stratified by HLA mismatch level 1&2 v 3&4.

The KDRI score was not associated with allograft loss, either as a continuous score or using the categorical D1-D4 risk groups [**Table 2**; **Figure 2A**]. The 2012 Watson KDRI was also not associated with allograft loss. On analysis of individual donor risk factors, the number of donors with history of diabetes or AKI were too small to allow meaningful analysis. Only donor height had a significant association with graft loss, though the hazard ratio was close to 1.0. There was no association of this outcome with other variables – donor age, sex, ethnicity, CMV status, history of hypertension, smoking, cause of death and eGFR at offer.

**Table 2:** Survival analysis for time to death-censored allograft failure based on univariate factors for donors and recipient donor characteristics.

Donor characteristics				
	Univariate analysis		Multivariate analysis	
	HR (95% CI)	p-value	HR (95% CI)	p-value
Donor sex Female (401) Male (507)	ref 0.91 (0.73-1.14)	ns		
Donor age (per year)	1.00 (0.99-1.01)	ns	1.01 (1.00-1.01)	0.056
Donor height	0.99 (0.98-1.00)	ns	0.99 (0.98-1.00)	0.01
Donor weight	1.00 (0.99-1.00)	ns		
Donor ethnicity White ethnic group (875) Minority ethnic group (33)	Ref 1.4 (0.87-2.54)	ns		
Donor eGFR	1.00 (1.00-1.00)	ns		
Donor CMV No (597) Yes (311)	Ref 0.98 (0.77-1.23)	ns		
Donor hypertension No (844) Yes (64)	Ref 1.14 (0.74-1.23)	ns		
Hospital stay (days)	0.99 (0.96-1.04)	ns		
Donor history of smoking No (554) Yes (354)	Ref 1.00 (0.80-1.26)	ns		
Cause of death Cardiovascular/Stroke (557) Trauma (232) Other (119)	Ref 0.96 (0.74-1.23) 1.08 (0.78-1.50)	ns		
Watson DRI	1.17 (0.57-2.38)	ns		
UK KDRI	1.15 (0.80-1.67)	ns		
UK KDRI D1 (577) D2 (230) D3 and 4 (101)	Ref 1.08 (0.84 -1.40) 1.03 (0.71-1.49)	ns		
Donor-recipient age difference (per year)	1.00 (0.99-1.00)	ns		
Recipient and transplant characteristics				
Recipient age (per year)	1.05 (1.02-1.08)	<0.001	1.05 (1.03-1.08)	<0.001
Recipient ethnicity White ethnic group (657) Minority ethnic group (251)	Ref 1.34 (1.09-1.75)	0.01	Ref 1.28 (1.01-1.63)	0.04
Primary renal disease Familial (116) Glomerular (155) Tubulointerstitial (233) Other (118) Unknown (286)	Ref 1.13 (0.75-1.70) 1.01 (0.69-1.48) 0.88 (0.56-1.38) 0.94 (0.65-1.36)	ns		
Pre-emptive transplant Yes (199) No (709)	Ref 1.38 (1.04-1.84)	0.02	Ref 1.38 (1.04-1.81)	0.02
Transplant year	1.00 (0.97-1.03)	ns		
Calculated reaction frequency	1.00 (1.00-1.00)	ns		
Cold ischaemic time	0.99 (0.97-1.02)	ns		
HLA mismatch Level 1 (100) Level 2 (593) Level 3 (182) Level 4 (33)	Ref 1.55 (1.01-2.36) 2.03 (1.29-3.20) 2.50 (1.36-4.62**)**	0.013<0.001<0.01	Ref 1.44 (0.94-2.23) 1.92 (1.19-3.11) 2.40 (1.26-4.58)	0.09 0.007 0.007

All factors were subsequently tested to establish a multi-variate model maximising the Akaike Information Criterion. Numbers in brackets represent number of patients at risk for categorical variables.

Recipient factors associated with worse graft survival were higher recipient age, minority ethnic groups, patients on dialysis at the time of transplant and higher level of HLA mismatching. The risk associated with age was non-linear; older recipients had an increased risk of transplant failure compared to younger recipients (spline model, **Figure S3**). Recipient factors not associated with graft loss included primary renal disease, year of transplant, calculated reaction frequency and cold ischaemic time. The proportion of patients who were highly sensitised or had prolonged cold ischaemic times was small (>80% sensitised: 3%; >24 hours cold ischaemic time: 6%).

**Figure 3:**
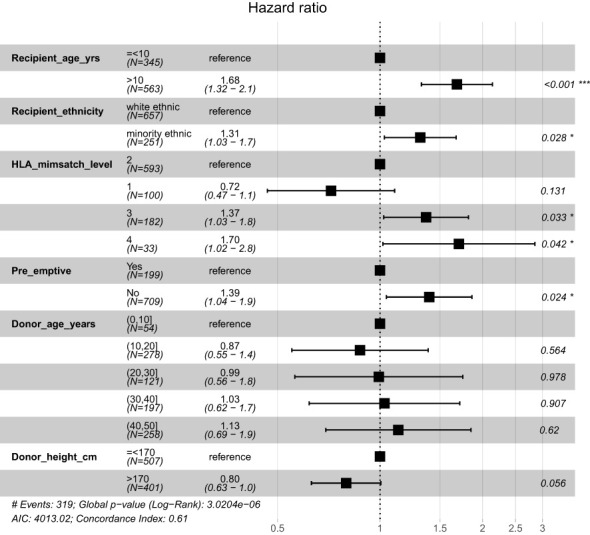
Forrest plot of multi-variate model with continuous variables split into categories to aid visualisation. *p<0.05; ***p<0.001.

In the final multi-variable model, all variables significant at univariate level were included. Additionally, donor age was included as it explained some of the outcome risk (model with the highest AIC). Recipients of ethnic minorities had a 29% increased risk and patients who were not pre-emptively transplanted had a 38% higher risk of graft loss. Patients who had Level 3 and 4 HLA mismatches were 1.9x and 2.4x more at risk of graft loss, compared to the Level 1 mismatch group respectively [**Figure 2B**]. Results of multi-variable analysis are presented in a forest plot [**Figure 3**].

**Figure 2C** shows the risk of all-cause allograft failure stratified by KDRI (D1 vs D2-4) and HLA level mismatch (Level 1-2 vs Level 3-4). HLA mismatch modifies the risk of allograft failure within the KDRI risk groups. Patients with Level 1-2 HLA mismatches had a median survival of >17 years, regardless of KDRI. Patients with Level 3-4 HLA mismatches had worse outcomes, even with transplants from D1 donors (median survival 16.9 years, p=0.002). Patients with both high risk donors and poor HLA match had worst outcomes (median survival 10.1 years, p=0.002).

### Sensitivity analysis

There were no differences in the donor characteristics for the patients who had graft failure in the first 30 days post-transplant [**Table S2**]. There was also no difference in graft survival for transplants performed before and after the change in kidney allocation (transplant year 2006 and later versus before 2006, p=0.9). There was no interaction between recipient age and HLA mismatch levels. The risk of graft failure according to recipient age was only significant in the first 10 years post-transplant (time-dependent survival analysis, **Table S3**). The risk of graft loss increased with each HLA mismatch (0-6 HLA-A/B/DR mismatch, adjusted HR 1.2 95% CI 1.1-1.4, p<0.001). We also reanalysed graft failure with death as a competing risk and the results were consistent [**Supplementary Table S4**]. To confirm, KDRI groups were also not significant using competing risk analysis (p>0.5 for D2 and D3-4 versus D1).

## Discussion

As kidney allocation systems incorporate donor risk indices into allocation policies, it is important to assess whether the scores are applicable to all subgroups of patients including paediatric recipients. We applied the UK-KDRI to a historical cohort of paediatric DBD kidney recipients from 2000-2014 and demonstrated that the usage of higher risk donors (D2-D4) increased whilst the level of HLA mismatching improved over time. The UK-KDRI did not predict death-censored allograft survival, whilst Level 3 and 4 HLA mismatches were associated with double the risk of graft failure compared to patient with Level 1 mismatches. Allocation schemes for paediatric kidney transplantation, therefore, need to balance both donor factors as well as HLA mismatching to better promote long-term allograft survival.

The UK-KDRI was originally derived from analysis of allograft failure in 7628 first adult kidney only donors and recipients with follow up of 5 years (12). Results were highly significant with a C-statistic of 0.64 and there was a high attrition of transplants within the first 30-days in the D2-D4 groups. The discriminative performance is moderate and comparable to other adult donor risk indices (for example US KDRI) but will misclassify 36% of the population, particularly within certain patient groups, such as the extremities of donor ages (>50 years and<18 years) and (as demonstrated in this study) in paediatric recipients (10, 11, 16–18). Similarly, Montgomery et al. reanalysed 9295 paediatric recipients from the UNOS database and showed that the US KDRI performed less well with C-statistic of 0.57 and a new paediatric specific KDRI with different variables (donor age was common to both models) improved prediction with C-statistic of 0.61 (19). The aim of our study was to validate the UK-KDRI rather than derive a new model. Notwithstanding the smaller number of patients, the UK-KDRI was not significantly associated with graft survival which suggests that the magnitude of effect was not as apparent in children.

Whilst the UK-KDRI allocates a subset of points in the UK Kidney Offering Scheme, the actual acceptance of individual kidneys is decided by local clinical team members. The historical decline rate of offers to paediatric recipients has been steady between 36-48% between 2011-2022, and is comparable to the decline rate for adult recipients (average 50% for standard criteria donors) (20). The main reasons for paediatric declines were ‘donor poor health or cause of death’ (75%) (21). 82% of declined offers were subsequently transplanted, mainly into adult recipients with 3-year allograft survival of 94%. Declined transplants which were subsequently transplanted into other paediatric recipients had lower allograft survival of 82% though the numbers were small and statistical analysis was not significant (21). Our results highlight the need for further studies which can identify factors (both donor quality as well as decision making behaviours) which can promote acceptance of suitable donors for transplant recipients.

Many factors are considered when accepting kidney donor offers and the decision to decline has to be weighed against the risks of continuing/commencing dialysis (5, 22). Whilst historically, waiting times for paediatric patients have been relatively short, any increase in waiting times for paediatric recipients will change the risk-benefit of accepting or declining offers. Schaapherder et al. used the paired nature of kidneys which were subsequently transplanted to different recipients as a control for donor quality (23). They found a smaller effect than expected of donor quality on graft loss at 1- and 10- years post-transplant. Furthermore, donors which were previously considered to be marginal have increasingly been used successfully to expand the donor pool in adults. Outcomes of DCD transplants, with careful donor selection, have equivocal outcomes compared to DBD donors, and these findings have also been reproduced in paediatric studies with early outcomes (24–27). Additionally, donors with acute kidney injury did not adversely affect recipient graft survival when adjusted for recipient factors, even when stratified by stage of acute kidney injury (28).

The effect of donor age was marginal in our study compared to results from studies in adult recipients (29). Nonetheless, more recent studies in adults have shown comparable graft survival outcomes using donors >65 years and >70 years (Pruett et al. and Echterdiek et al. respectively), when analysis was performed in a more contemporaneous era (after year 2000) (30, 31). In paediatrics studies, Chesnaye et al. also reported similar graft survival outcomes for donors up to 50 years in an analysis of 4686 transplants from the ESPN/ERA-EDTA registry (32). This was corroborated by analysis of 9209 paediatric transplants from the Collaborative Transplant Study (33). Better biomarkers are therefore required to assess biological age rather than chronological age. During the study period, the maximum donor age for DBD allocation to paediatric recipients was 50 years, and this was increased in September 2021 to 60 years.

Whilst our study did not show a statistical significance for donor quality variables, higher levels of HLA mismatch were clinically and statistically significant for higher risk of transplant failure. The main cause of transplant failure was also reported to be due to ‘rejection’. It is tempting to speculate that once a certain threshold of functioning donor nephron mass is achieved, chronic alloimmune injury from HLA mismatches becomes the main driver of transplant failure (34, 35). Whilst previous studies showed no effect of HLA matching when donors were<35 years of age, allograft outcome was only reported up to 5 years follow-up and the effect of HLA mismatching may become apparent with longer follow-up times (34, 36). Here again, caution is needed, as patients with rarer HLA types will have less access to well matched donors and patients with high levels of anti-HLA antibody sensitisation will have less opportunity to find well-matched HLA type combinations.

The main strength of our study is the completeness of data and long-term follow-up through to adult care made possible by NHSBT. Post-transplant management of this cohort will likely reflect UK practices, which may reduce generalisability to other populations. Results should be interpreted within the limits of the donor and recipient population and not extrapolated to very high-risk donors which were not well-represented. Other limitations are inherent in the statistical models utilised. Cox-proportional hazards models assume that variables are independent, and therefore interactions between donor factors are not implicit in the model. Latent variables not recorded as donor factors are also not considered.

Overall, kidney allocation schemes face a balancing act of matching longevity of the allografts to the expected gain in quality life years in the recipient, whilst not prejudicing against any specific patient groups (6, 12). Although we did not show any statistical significance of any donor variables, we are not arguing for their non-significance but rather highlighting that when models are developed in kidney transplantation using only adult patients, the same prediction performance might not be obtained in paediatric populations. As prediction models become more complex and sophisticated and particularly where those models are then used in organ allocation, we advocate that the entirety of the patient population, adults and children, be included in the model development.

## Data availability statement

The original contributions presented in the study are included in the article/**Supplementary Material**, further inquiries can be directed to the corresponding author.

## Ethics statement

Ethical review and approval was not required for the study on human participants in accordance with the local legislation and institutional requirements. Written informed consent from the participants’ legal guardian/next of kin was not required to participate in this study in accordance with the national legislation and the institutional requirements.

## Author contributions

JK, RC, SM, JD and AW contributed to conception and design of the study. JK performed the statistical analysis. JK wrote the first draft of the manuscript. JK, RC, BR, SM, VK and JD wrote sections of the manuscript. All authors contributed to the article and approved the submitted version.
