# Supplementary material for: The UK kidney donor risk index poorly predicts long-term transplant survival in paediatric kidney transplant recipients
Source: Front Immunol. 2023 Jun 2;14:1207145. doi: 10.3389/fimmu.2023.1207145 (PMC10275486; doi:10.3389/fimmu.2023.1207145)
Supplement: Supplementary file 1 [file DataSheet_1.pdf]

Supplementary Material

Table S1: Recipient characteristics of first paediatric DBD kidney only, from 2000-2014, left-censored for graft survival >30 days post-transplant.

Table S2: Characteristics of donors excluded (due to graft failure within 30 days post-transplant) were not significantly different to donors included in the analysis.

Table S3: Multi-variate model using recipient age as a time-dependent variable, divided into periods 0-10 years post-transplant and >10 years post-transplant.

Table S4: Competing risk analysis (Fine-Gray method) for primary outcome of graft failure with death as a competing risk.

Figure S1: Details of missing data: Proportion of missing data for donor (A) and recipient (B) variables. C) Visualisation of missing data showing missing data as random.

degfr: donor eGFR at retrieval, LAST_UO: urine output in the final hour, Smoker: current or past cigarette smoker, D_HT: donor hypertension, hosp_stay: length of hospital stay, D/RHEIGHT: donor/recipient height, D_DM: donor history of diabetes mellitus, D/RETHNIC: ethnicity, D/RSEX: sex, D/RAGE: age, D/RBG: donor blood group, D/RWEIGHT: donor weight, DGF: delayed graft function, CIT_HRS: cold ischaemic time (hours), Pre-Emp: pre-emptive transplantation, PRD: primary cause of kidney failure, TX_YR: year of transplant, A/B/DRMM: A/B/DR HLA mismatch, HLA_GRP: NHSBT HLA levels

Figure S2: Cumulative incidence of graft failure based on cause of graft failure.

Figure S3: Log hazard of recipient age for all-cause allograft failure is non-linear

Recipient Characteristic	N = 908[1]
SEX	
**F**	377 (42%)
**M**	531 (58%)
AGE	12 (8, 16)
Blood Group	
**A**	297 (33%)
**AB**	51 (5.6%)
**B**	134 (15%)
**O**	426 (47%)
ETHNIC	
**Minority ethnic group**	251 (28%)
**White ethnic group**	655 (72%)
**Unknown**	2 (<1%)
Primary renal disease	
**Glomerular**	155 (17%)
**Tubulointersitital**	233 (26%)
**Other**	118 (13%)
**Familial**	116 (13%)
**Unknown**	286 (31%)
Dialysis status at time of transplant	
No	199 (22%)
Yes	703 (78%)
Unknown	6 (<1%)
Calculated reaction frequency at time of transplant (%)	0 (0, 35)
Cold ischaemic time (hours)	16.1 (13.8, 18.9)

Table S1: Recipient characteristics of first paediatric DBD kidney only, from 2000-2014, left-censored for graft survival >30 days post-transplant

Donor characteristic	Allograft failure <30 days (n=41)	Allograft failure ≥30 days (n=908)	p-value
Age (years)	33 (14, 45)	31 (17, 41)	ns
Sex			ns
Female	15 (37%)	401 (44%)	
Male	26 (63%)	507 (56%)	
Ethnicity			ns
White ethnic group	38 (93%)	871 (96%)	
Minority ethnic groups	3 (7%)	33 (4%)	
Height (cm)	170 (159, 177)	170 (161, 178)	ns
Weight (kg)	70 (55, 80)	68 (60, 80)	ns
eGFR at offer (ml/min/1.73m^2)	97 (69, 141)	103 (80, 134)	ns
History of hypertension (yes)	2 (5%)	64 (7%)	ns
CMV status (positive)	13 (32%)	311 (35%)	ns
Hospital stay (days)	1 (1, 2)	1 (1, 3)	ns
Adrenaline use (yes)	9 (22%)	131 (14%)	ns
History of diabetes (yes)	1 (2.5%)	11 (1%)	ns
History of smoking (yes)	11 (27%)	354 (40%)	ns
Cause of death			ns
Cardiovascular or stroke	29 (71%)	557 (61%)	
Trauma	8 (19%)	232 (26%)	
Other	4 (10%)	119 (13%)	

Table S2: Characteristics of donors excluded (due to graft failure within 30 days post-transplant) were not significantly different to donors included in the analysis.

Risk factor	Adjusted HR (95% CI)	P value
Recipient age (per year)		
<10 years post-transplant	1.08 (1.05-1.11)	<0.001
>10 years post-transplant	0.99 (0.94-1.04)	ns
Recipient ethnicity		
White ethnic group (657)	Ref	<0.05
Minority ethnic group (251)	1.29 (1.02-1.65)	
Pre-emptive transplant		
Yes (199)	Ref	<0.05
No (709)	1.38 (1.02-1.81)	
Donor age (per year)	1.00 (1.00-1.02)	0.05
Donor height (per cm)	0.99 (0.98-1.00)	<0.05
HLA mismatch		
Level 1	Ref	
Level 2	1.45 (0.94-2.22)	0.09
Level 3	1.95 (1.20-3.15)	<0.001
Level 4	2.43 (1.27-4.63**)**	<0.01

Table S3: Multi-variable model of graft loss using recipient age as a time-dependent variable, divided into periods 0-10 years post-transplant and >10 years post-transplant.

**Risk factor**	**Adjusted HR (95% CI)**	**P value**
Recipient age (per year)	1.05 (1.03-1.08)	<0.001
Recipient ethnicity		
White ethnic group (657)	Ref	<0.05
Minority ethnic group (251)	1.29 (1.01-1.65)	
Pre-emptive transplant		
Yes (199)	Ref	<0.05
No (709)	1.38 (1.03-1.85)	
Donor age (per year)	1.01 (1.00-1.02)	<0.05
Donor height (per cm)	0.99 (0.98-1.00)	<0.05
HLA mismatch		
Level 1	Ref	
Level 2	1.40 (0.91-2.16)	0.13
Level 3	1.87 (1.15-3.02)	<0.05
Level 4	2.39 (1.25-4.55)	<0.001

Table S4: Competing risk analysis (Fine-Gray method) for primary outcome of graft failure with death as a competing risk.

A)

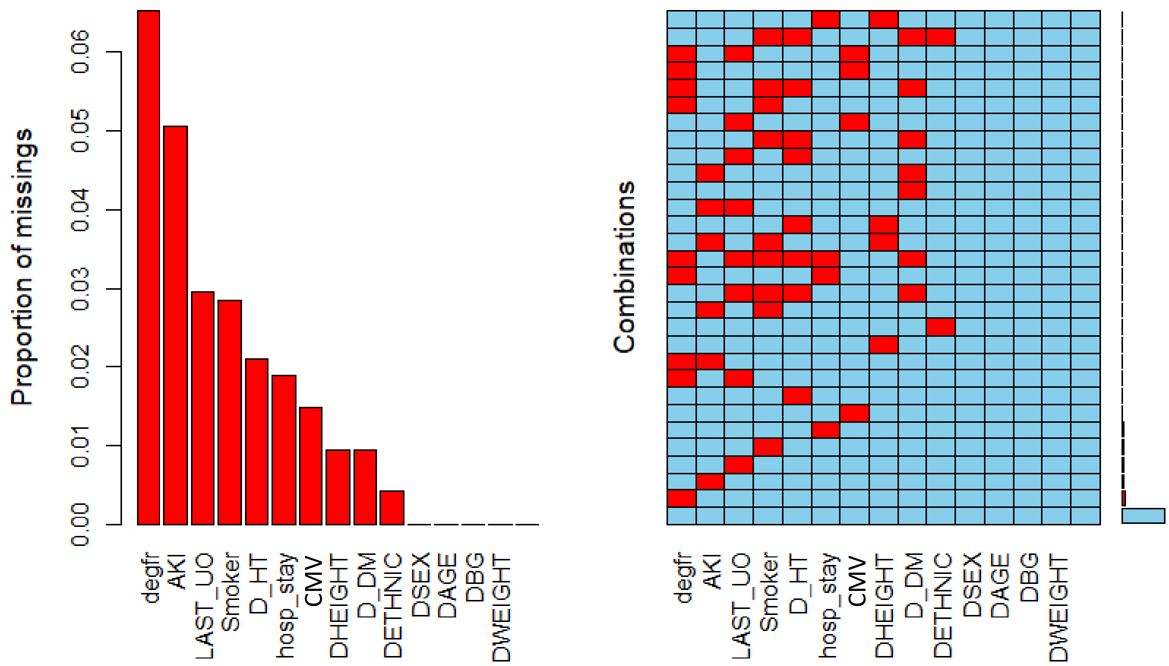

Proportion of missings
0.00
0.01
0.02
0.03
0.04
0.05
0.06
degfr
AKI
LAST_UO
Smoker
D_HT
hosp_stay
CMV
DHEIGHT
D_DM
DETHNIC
DSEX
DAGE
DBG
DWEIGHT
Combinations

B)

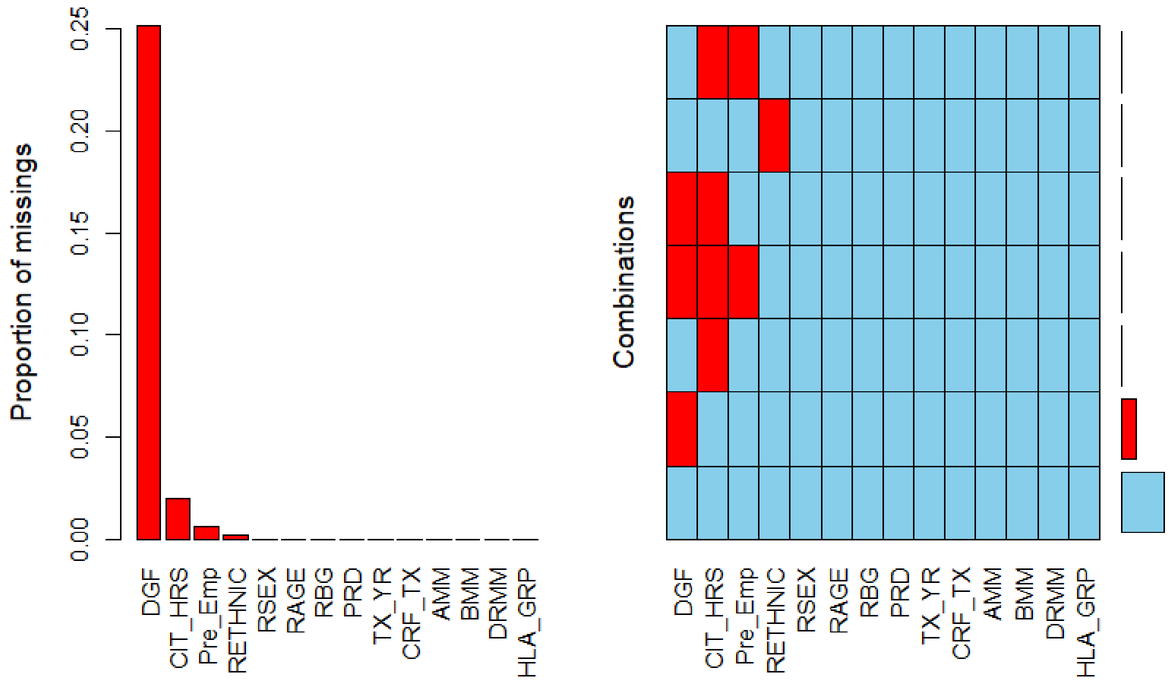

Proportion of missings
0.00
0.05
0.10
0.15
0.20
0.25
DGF
CIT_HRS
Pre_Emp
RETHNIC
RSEX
RAGE
RBG
PRD
TX_YR
CRF_TX
AMM
BMM
DRMM
HLA_GRP
Combinations

C)

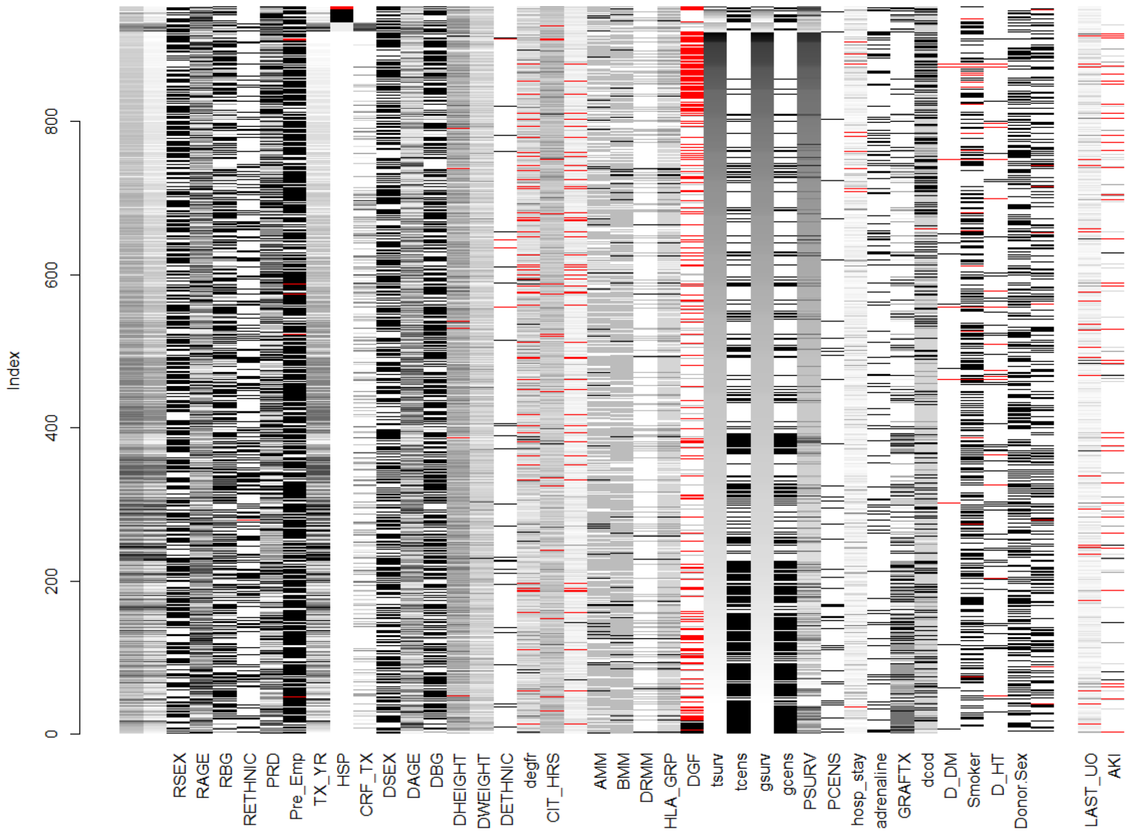

Figure S1: Details of missing data: Proportion of missing data for donor (A) and recipient (B) variables. C) Visualisation of missing data showing missing data as random.

degfr: donor eGFR at retrieval, LAST_UO: urine output in the final hour, Smoker: current or past cigarette smoker, D_HT: donor hypertension, hosp_stay: length of hospital stay, D/RHEIGHT: donor/recipient height, D_DM: donor history of diabetes mellitus, D/RETHNIC: ethnicity, D/RSEX: sex, D/RAGE: age, D/RBG: donor blood group, D/RWEIGHT: donor weight, DGF: delayed graft function, CIT_HRS: cold ischaemic time (hours), Pre-Emp: pre-emptive transplantation, PRD: primary cause of kidney failure, TX_YR: year of transplant, A/B/DRMM: A/B/DR HLA mismatch, HLA_GRP: NHSBT HLA levels

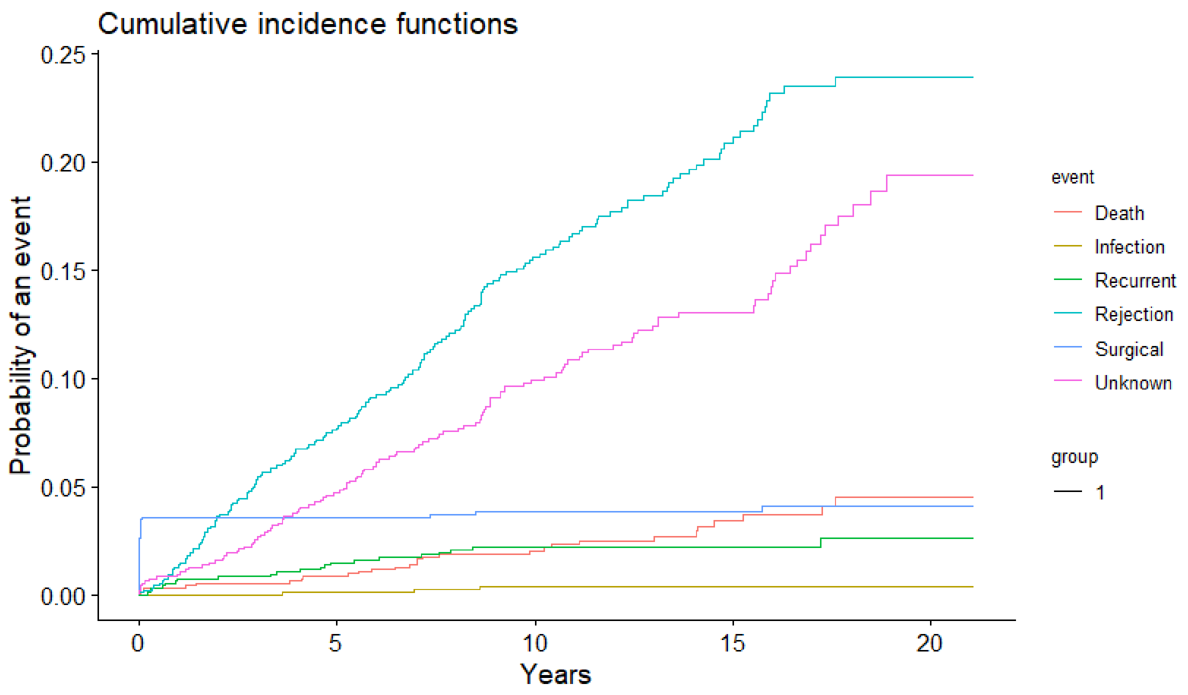

Figure S2: Cumulative incidence of graft failure based on cause of graft failure.

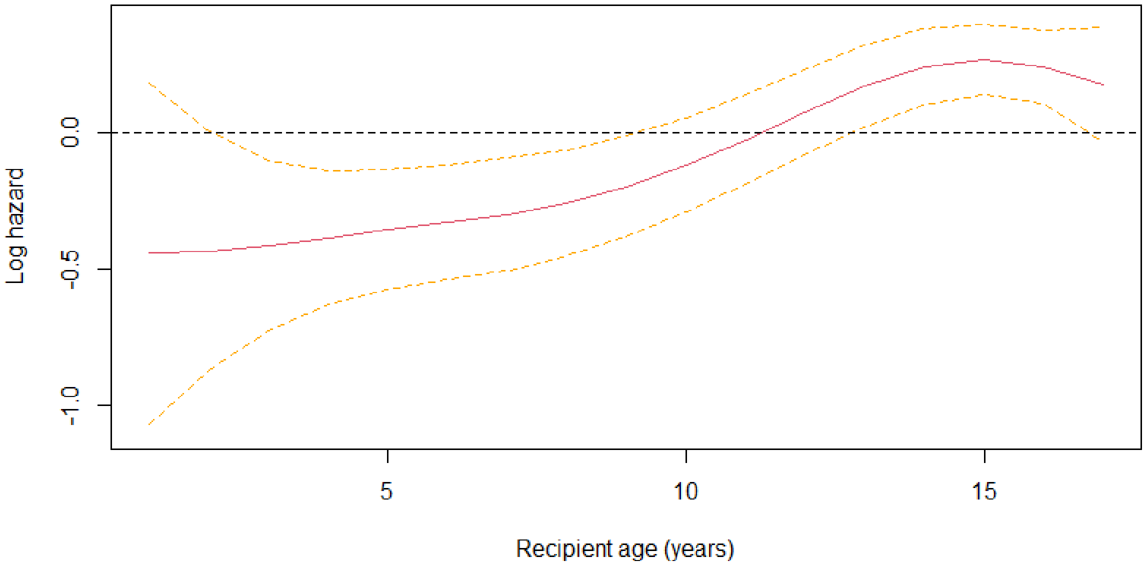

Figure S3: Log hazard of recipient age for all-cause allograft failure is non-linear
